# Differential Responses of *Salmonella enterica* Typhimurium, *S. enteritidis*, and *S. infantis* to Chlorine Dioxide In Vitro: Impacts on Growth and Biofilm Development

**DOI:** 10.3390/microorganisms14051058

**Published:** 2026-05-08

**Authors:** Inkar Castellanos-Huerta, Jacob Lum, Guillermo Romero, Aaron Forga, Billy M. Hargis, Danielle Graham

**Affiliations:** 1 Department of Poultry Science, University of Arkansas, Fayetteville, AR 72701, USA; icastell@uark.edu (I.C.-H.); ajforga@uark.edu (A.F.); bhargis@uark.edu (B.M.H.); 2 Ozark Avian Research LLC, Gravette, AR 72736, USA; jacob@ozarkavianresearch.com; 3 Kemin Industries Inc., Des Moines, IA 50317, USA; guillermo.romero@kemin.com

**Keywords:** chlorine dioxide, biofilm, *Salmonella* Typhimurium, *Salmonella* Enteritidis, *Salmonella* Infantis

## Abstract

*Salmonella enterica* is a significant Gram-negative bacterium possessing over 2500 serovars capable of affecting both animals and humans and disseminating widely due to its adaptability, genetic diversity, and ability to form biofilms. Different serovars, such as *S. enterica* Typhimurium (ST), Enteritidis (SE), and Infantis (SI), display varying traits and survival strategies in harsh environments. Biofilms, composed of proteins, lipids, and DNA, enable bacteria to survive stresses such as pH changes, nutrient shortages, temperature fluctuations, and disinfectants. Evaluating disinfectants on inert surfaces is crucial for understanding their effectiveness and impact on poultry. This study assessed the efficacy of chlorine dioxide (ClO_2_) disinfectant against ST, SE, and SI growth, biofilm formation, and biofilm removal at varying concentrations in vitro. Results showed serotype-dependent and condition-specific responses, with SE and SI being more affected than ST, which may be associated with differences in oxidative stress response mechanisms, highlighting the need for tailored disinfection protocols.

## 1. Introduction

*Salmonella enterica* is a Gram-negative bacterium that is adaptable to various hosts and environments, enhancing its survival and infection ability in humans, particularly in serovars Typhimurium (ST), Enteritidis (SE), and Infantis (SI) [1,2,3,4,5,6,7]. *Salmonella* survives on surfaces by forming a biofilm with a complex extracellular polymeric substance (EPS) matrix, which includes channels for nutrient transport, communication, and waste removal [4,5,6,7,8,9,10,11,12]. Biofilm formation begins with reversible adhesion mediated by hydrophobic or electrostatic forces. Bacteria produce an extracellular matrix, forming microcolonies that develop into a protective three-dimensional biofilm. This boosts their survival in extreme conditions and makes them up to 1000 times more resistant to disinfectants than planktonic bacteria [9,10,11,12,13,14,15,16].

Chlorine disinfectants such as chlorite ions (ClO_2_^−^) and chlorine dioxide (ClO_2_) prevent the growth of microbes and biofilms in the water distribution system. ClO_2_, a pre-oxidant and primary disinfectant for the treatment of drinking water, is used for its ability to maintain activity despite organic matter, its effectiveness across a wide pH range [17], and its lack of formation of harmful organohalogen byproducts. However, high temperature could reduce its concentration to undetectable levels [18]. ClO_2_ effectively eliminates heterotrophic bacteria from pipe surfaces, achieving log reductions of 0.52–1.36 at 0.5 ppm, whereas low concentrations of chlorite (0.1 ppm) showed minimal effects (log reductions of 0.20–0.34) [19]. Although toxic to rats at 170–182 ppm Lethal Dose 50% (LD50), chlorine disinfectants are considered safe for water treatment at concentrations of 0.5–2 ppm [19,20,21]. In instances of high contamination in pipe water lines, “shock” treatments at 40–80 ppm are suggested to eliminate biofilm and pathogens [22,23]. Understanding the ability of different *Salmonella enterica* serotypes to adapt to environmental conditions, including exposure to disinfectants, is critical to optimizing disinfection and sanitation protocols. ST, SE, and SI exhibit notable variability in their responses to ClO_2_, particularly in terms of transcriptomic expression levels of genes related to oxidative stress response, biofilm formation, energy metabolism, and membrane repair and membrane transport [24,25,26]. The purpose of this study was to evaluate the effect of a commercially available ClO_2_ disinfectant on the in vitro growth of ST, SE, or SI, as well as its impact on biofilm formation and removal at increasing concentrations (0–496 ppm free ClO_2_).

## 2. Materials and Methods

### 2.1. Bacterial Samples

Aliquots (preserved in 10% glycerol and stored at −80 °C) of *S. enterica* ST, SE (nalidixic acid and novobiocin-resistant strains) [3], and SI (field strain) from the Poultry Health Laboratory at the University of Arkansas were used in the present assessment. For the assay, 10 μL of ST, SE, or SI from an aliquot was used to inoculate 10 mL of tryptic soy broth (TSB; MilliporeSigma, Burlington, MA, USA) for 12 h at 37 °C without agitation until they reached an optical density (OD) of 0.6 at 600 nm. OD was measured using a microplate reader (SpectraMax iD5, Molecular Devices, San Jose, CA, USA). The samples were evaluated to determine colony-forming units per milliliter (CFU/mL) using Xylose Lysine Tergitol-4 agar (XLT-4 agar, MilliporeSigma, Burlington, MA, USA), incubated aerobically at 37 °C overnight, and Log_10_ CFU/mL values were confirmed by plating.

### 2.2. Assessment of the Bactericidal Effect and Impact of ClO_2_ on Biofilm Development in Salmonella enterica

A commercial ClO_2_ disinfectant (PRO-OXINE^®^ AH (Kemin Industries, Inc., Norman, OK, USA) was used in this study. This two-component chlorine dioxide-generating system (sodium chlorite and an acid activator) was activated according to the manufacturer’s instructions to produce ClO_2_ in situ. ClO_2_ concentrations were quantified prior to each experimental use using a DPD colorimetric method specific for chlorine dioxide. Concentrations were selected based on previously reported effective ranges, including values up to 80 ppm, considered acceptable for practical applications [23], as well as values above 60 ppm, since 50 ppm are not effective in achieving an antagonistic effect against *Salmonella* spp. [27]. Individual cultures of 10^6^ colony-forming units (CFU)/200 μL per well of *S. enterica* serotypes ST, SE, and SI in TSB (MilliporeSigma, Burlington, MA, USA) were seeded into sterile non-coated 96-well polystyrene U-shaped plates (Falcon, NY, USA). Cultures were treated with three different concentrations of free ClO_2_, i.e., 62, 124, and 248 ppm, and incubated at 37 °C for 12 h. Controls not treated were included during all the experiments. Post-incubation, briefly, the samples were centrifuged at 12,000 rpm (≈1.3 × 10^5^ g) for 5 min at room temperature and washed with physiologic saline solution (PSS), subsequently resuspended in 100 μL of TSB, and immediately read at 600 nm in a sterile flat-bottom 96-well plate to determine their OD and CFU/mL with a standard curve. To establish the standard curve for each bacterial serotype, three independent cultures (biological replicates) of *S. enterica* serotypes ST, SE, and SI were incubated overnight at 37 °C in 10 mL of TSB. The cultures were then centrifuged at 12,000 rpm (≈1.3 × 10^5^ g) for 5 min at room temperature and resuspended in 10 mL of fresh TSB. For each biological replicate and serotype, 100 μL (*n* = 7) were subjected to a serial tenfold dilution in a sterile flat-bottom 96-well plate. Samples diluted 1:8 were used for absorbance measurements with a microplate reader at 600 nm. Concurrently, Log_10_ cells/mL were confirmed by serial dilution and drop plating on XLT-4 agar (MilliporeSigma, Burlington, MA, USA).

The assessment of biofilm formation was conducted according to Latasa, C. et al., 2012 [28], with the following modifications. Briefly, each U-bottom plate was carefully inverted and struck on the bottom to remove the liquid portion of the bacterial culture without disturbing the biofilm that was deposited on the plates’ surfaces, rinsed three times with PBS, fixed with 100% methanol, rinsed again, stained with 1% crystal violet, washed once more with tap water, and resuspended in 90% ethanol for absorbance measurement at 600 nm using a microplate reader. For each *Salmonella* serotype, three biological replicates were prepared. From each, seven technical replicates (*n* = 7) were obtained, and the experiment was independently repeated three times.

### 2.3. The Elimination of Biofilm Formation in Cultures of Salmonella enterica Utilizing Chlorine Dioxide (ClO_2_)

This treatment involved individual cultures with 10^6^ CFU/200 μL of serotypes ST, SE, and SI in TSB medium in 96-well plates. After 12 h at 37 °C [29], the medium was removed from the bacterial culture by inversion, being careful not to harm the biofilm, and biofilms were treated with four different concentrations of free ClO_2_ at 62, 124, 248, and 496 ppm for 15 min at room temperature. Control groups received no treatment. Results are shown as OD600, evaluated with 1% crystal violet staining as previously described [28]. Once again, three biological replicates were prepared for each Salmonella serotype. From each, seven technical replicates (*n* = 7) were obtained, and the experiment was independently repeated three times.

### 2.4. Determination of Fold Change, Percentage Change, and Statistical Analysis

Log_10_ CFU/mL and OD600 were analyzed to determine percent and fold change relative to non-treated controls using the following formulas:

% change = [(treatment value − non-treated control value)/non −treated control value] × 100

Fold change=Treatment value/non-treated control value

These formulas were applied independently to Log_10_ CFU/mL and OD600 values. For each serotype and ClO_2_ concentration, percentage change and fold change were reported, all relative to the respective non-treated control. Differences between means were analyzed using one-way Analysis of Variance (ANOVA) followed by Tukey’s post hoc test to identify differences between treatments, with a significance threshold set at *p* < 0.05. The statistical analysis was performed using PRISM software (GraphPad, v.10).

## 3. Results

### 3.1. Effect of ClO_2_ on ST, SE, or SI Growth In Vitro

To evaluate the effect of ClO_2_ on ST, SE, and SI growth in vitro, cultures were incubated with concentrations of free ClO_2_ (62, 124, and 248 ppm) for 12 h at 37 °C. Log10 CFU/mL was determined after 12 h. To evaluate the effect of ClO_2_ on ST, SE, and SI growth in vitro, cultures were incubated with free ClO_2_ at 62, 124, and 248 ppm for 12 h at 37 °C. Bacterial counts were determined as Log_10_ CFU/mL after incubation.

**Figure 1:**
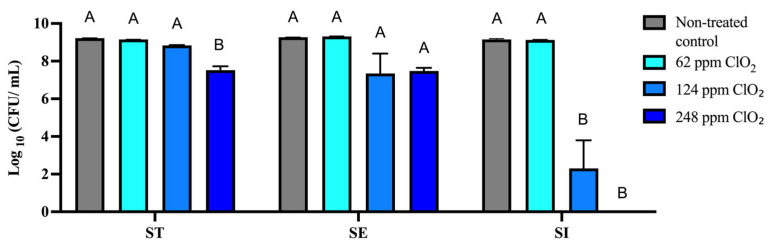
Effect of chlorine dioxide (ClO_2_) at 62, 124, and 248 ppm on the in vitro growth of *S. enterica* serovars Typhimurium (ST), Enteritidis (SE), and Infantis (SI), expressed as Log_10_ CFU/mL after 12 h of incubation at 37 °C. Data are presented as mean ± SEM from three independent experiments. Different letters indicate statistically significant differences among ClO_2_ treatments within each serotype (*p* < 0.05), as determined by one-way ANOVA followed by Tukey’s multiple comparisons test.

In ST, no significant differences were observed among the non-treated control, 62 ppm treatments, and 124 ppm treatments (*p* > 0.05). However, exposure to 248 ppm significantly reduced bacterial counts compared with the non-treated control and the lower ClO_2_ concentrations (*p* < 0.05). In SE, ClO_2_ exposure did not significantly affect bacterial growth at any of the concentrations tested, as no significant differences were observed among the non-treated control, as well as the 62 ppm, 124 ppm, and 248 ppm treatments (*p* > 0.05). In SI, no significant difference was observed between the non-treated control and 62 ppm treatment (*p* > 0.05). In contrast, bacterial counts were significantly reduced at 124 and 248 ppm compared with the non-treated control and 62 ppm treatment (*p* < 0.05), with no significant difference between 124 and 248 ppm (*p* > 0.05) (Figure 1).

The analysis of CFU/mL values (Table 1) further supported the serotype-dependent response to ClO_2_ observed in Figure 1, with serovar SI showing the highest susceptibility under the tested in vitro conditions. Consistent with the reductions observed at 124 and 248 ppm, bacterial counts dropped markedly relative to the non-treated control. At 124 ppm, growth was detected at very low levels (2.02 × 10^2^ CFU/mL), corresponding to a 99.9% reduction (fold change = 0.01), whereas at 248 ppm, no colonies were detected, indicating inhibition below the detection limit. In contrast, serovar SE exhibited a different pattern. A slight increase in bacterial counts was observed at 62 ppm (+10.2%; fold change = 1.10), followed by a sharp reduction at higher concentrations, with reductions of 99.1% and 98.4% at 124 and 248 ppm, respectively (fold changes = 0.01 and 0.02). For serovar ST, the effect of ClO_2_ was more gradual, with a 14.4% reduction at 62 ppm (fold change = 0.86), a 58.6% reduction at 124 ppm (fold change = 0.41), and a pronounced 98.0% reduction at 248 ppm (fold change = 0.02). Overall, percentage reduction and fold-change values were calculated relative to the untreated control for each serotype using CFU/mL data. Although differences appear less pronounced on the Log_10_ scale (Figure 1), these correspond to substantial reductions in bacterial counts when expressed in CFU/mL (Table 1), particularly at higher ClO_2_ concentrations where bacterial growth approached or fell below detectable limits (Table 1).

### 3.2. Impact of ClO_2_ on ST, SE, or SI Biofilm Formation In Vitro

**Figure 2:**
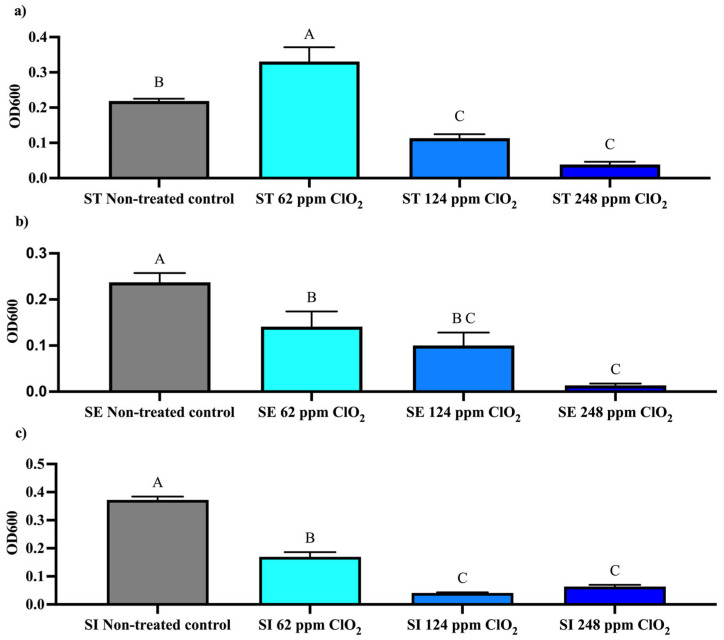
Effect of chlorine dioxide (ClO_2_) on biofilm formation of *S. enterica* serovars. (**a**) Typhimurium (ST), (**b**) Enteritidis (SE), and (**c**) Infantis (SI), quantified by crystal violet staining and expressed as OD_600_. Data are presented as mean ± SEM from three independent experiments. Different letters indicate significant differences among treatments within each serotype (*p* < 0.05), as determined by one-way ANOVA followed by Tukey’s multiple comparisons test.

**Figure 3:**
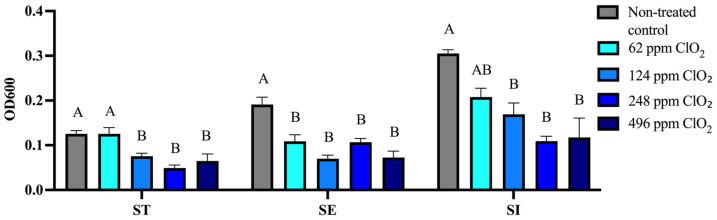
Effect of chlorine dioxide (ClO_2_) at concentrations of 62, 124, 248, and 496 ppm on the elimination of pre-formed biofilms of *S. enterica* serovars Typhimurium (ST), Enteritidis (SE), and Infantis (SI). Biofilms were exposed to ClO_2_ for 15 min, and residual biomass was quantified by crystal violet staining (OD_600_). Data are presented as mean ± SEM from three independent experiments. Different letters indicate statistically significant differences among treatments within each serotype (*p* < 0.05), as determined by one-way ANOVA followed by Tukey’s multiple comparisons test.

The effect of ClO_2_ on ST, SE, and SI biofilm formation was investigated using free ClO_2_ at concentrations of 62, 124, and 248 ppm. Biofilm was measured using 1% crystal violet at OD 600 nm. Biofilm formation by the ST serotype increased significantly (*p* < 0.05) at 62 ppm of ClO_2_ and decreased when the concentration was raised to 124 and 248 ppm compared to the ST control group (Figure 2a). On the other hand, SE biofilm formation decreased significantly (*p* < 0.05) at 62 and 124 ppm, and more markedly at 248 ppm, compared with the respective control groups (Figure 2b). Finally, serotype SI showed reduced biofilm formation (*p* < 0.05) at 62 ppm and was highly affected at 124 and 248 ppm of ClO_2_ (Figure 2c). Without treatment, ST, SE, and SI exhibited high biofilm formation, with SI showing the highest capacity, followed by ST and SE (Figure 2c and Figure 3).

Analysis based on OD600 values indicated that biofilm formation for serotype SI was the most susceptible to ClO_2_, exhibiting an 89% reduction (fold change = 0.11) at 124 ppm and an 82.93% reduction (fold change = 0.17) at 248 ppm. Similarly, serotype SE showed a marked, concentration-dependent reduction, with decreases of 40.55% (fold change = 0.59), 57.80% (fold change = 0.42), and 94.23% (fold change = 0.06) at 62, 124, and 248 ppm, respectively. Conversely, serotype ST showed an increase in biofilm formation at 62 ppm (+51.32%; fold change = 1.51), followed by a concentration-dependent reduction at 124 ppm (−48.03%; fold change = 0.52) and 248 ppm (−82.24%; fold change = 0.18) (Table 2).

### 3.3. Elimination of ST, SE, or SI Biofilms Pre-Formed In Vitro Utilizing ClO_2_

To assess the effectiveness of ClO_2_ in eliminating pre-formed biofilms of *S. enterica* serovars ST, SE, and SI, biofilms were exposed to free ClO_2_ at concentrations of 62, 124, 248, and 496 ppm for 15 min. Residual biofilm biomass was quantified by crystal violet staining and expressed as OD_600_.

In ST, exposure to ClO_2_ led to a progressive decrease in biofilm biomass with increasing concentration. A significant reduction was observed at 124, 248, and 496 ppm compared with the non-treated control (*p* < 0.05), while no significant difference was detected following treatment with 62 ppm. For SE, ClO_2_ treatment resulted in a marked reduction in biofilm biomass across all tested concentrations. Even at 62 ppm, a significant decrease was observed compared with the non-treated control (*p* < 0.05), with further reductions as the concentration increased. Similarly, for SI, biofilm biomass was significantly reduced following treatment with 124 ppm, 248 ppm, or 496 ppm ClO_2_ compared to the non-treated control (*p* < 0.05). Overall, these results indicate that ClO_2_ effectively reduces pre-formed biofilms in a concentration-dependent and serotype-dependent manner, with SE and SI showing greater susceptibility, whereas ST exhibits a comparatively more gradual response (Figure 3).

Analysis of OD_600_ values following ClO_2_ treatment across the evaluated serotypes indicated that the elimination of pre-formed biofilm varied quantitatively among the serotypes. In the case of serotype ST, no effect was observed at 62 ppm (−0.08%; fold change = 1.00), whereas reductions were observed at 124 ppm (−39.72%; fold change = 0.60) and 248 ppm (−60.70%; fold change = 0.39), followed by a smaller reduction at 496 ppm (−48.30%; fold change = 0.52). For serotype SE, a decrease in biofilm was observed at all concentrations evaluated, with reductions of 43.01% (fold change = 0.57), 63.42% (fold change = 0.37), 43.90% (fold change = 0.56), and 60.54% (fold change = 0.38) at 62, 124, 248, and 496 ppm, respectively. SI showed a smaller reduction at 62 ppm (−9.21%; fold change = 0.68), followed by more pronounced reductions at 124 ppm (−44.51%; fold change = 0.55), 248 ppm (−64.20%; fold change = 0.36), and 496 ppm (−64.45%; fold change = 0.39) (Table 3).

These findings suggest that the impact of ClO_2_ is contingent upon the serotype and the specific condition assessed. The results demonstrate improved effectiveness against planktonic cells during biofilm development, whereas eradication of pre-existing biofilm was less effective and showed greater variability across serotypes.

## 4. Discussion and Conclusions

Studies indicate that *S. enterica* biofilms pose a primary challenge for cleaning and disinfection in poultry processing plants and throughout live production, including hatcheries, farms, and transport vehicles [30]. As a result, the use of disinfectants with oxidizing activity (iodine and chlorine) or non-oxidizing agents (amphoteric compounds and quaternary ammonium compounds) [20] is highly recommended, as they offer a broad antimicrobial spectrum and can penetrate biofilm structures [31]. In the present study, the objective was to determine the effect of a ClO_2_-based disinfectant in contact with three serotypes of *S. enterica* (ST, SE, and SI) by evaluating the impact on the survival of planktonic cells in TSB medium, biofilm formation capacity in vitro, and, finally, the efficiency of ClO_2_ to remove biofilms produced by ST, SE, and SI in vitro. The results demonstrate a clear serotype-dependent and condition-specific response, rather than a strictly linear or dose-dependent behavior, highlighting the complexity of ClO_2_ efficacy against *Salmonella*.

The present study indicates that 62 ppm ClO_2_ did not significantly affect planktonic growth in vitro. However, higher concentrations (124 and 248 ppm) resulted in substantial reductions in ST and SI, particularly for SI, whereas no statistically significant differences were detected in SE across the concentrations tested. Although these Log_10_ reductions appear moderate (Figure 1), they correspond to significant decreases in CFU/mL (Table 1), highlighting the importance of interpreting microbial inactivation from both logarithmic and linear perspectives. Quantitative data indicated reductions in ST and SE, although no statistically significant differences in SE were observed across treatments, with more pronounced effects on SI (fold changes approaching 0.01 or lower), implying differential sensitivity to elevated concentrations beginning at 124 ppm. A similar differential response to ClO_2_ among serotypes has been reported previously in antimicrobial activity studies [32].

Variability among serotypes indicates that disinfectant efficacy depends on concentration, environmental conditions, and the physiological state of the bacteria. For example, Banach et al. demonstrated that the efficacy of ClO_2_ and other sanitizers is strongly influenced by factors such as organic load, temperature, and bacterial adhesion, which often override intrinsic differences in susceptibility [33]. These results indicate that the diverse responses seen in this study arise from complex interactions between the disinfectant and the biological system, rather than straightforward dose–response effects. This contrasts with earlier findings for gas applications of ClO_2_ at 20 ppm and 80 ppm, which reduced SE and *Salmonella* Gallinarum (SG) growth by 4 logs [34]. Interestingly, it was observed that at low concentrations of ClO_2_ (62 ppm), SE serotype exhibited an increase in growth of approximately 10.2%, which was unexpected. A similar phenomenon has been observed in other experiments, where low concentrations of chlorine-based disinfectants facilitate the regrowth or increased proliferation of potentially resistant microorganisms during treatment [35,36]. Although a formal determination of MIC for ClO_2_ was not conducted in the present study, previous reports have established that ClO_2_ is effective against *S. enterica* at concentrations as low as 20 ppm under optimal conditions; furthermore, it has been observed that higher concentrations (40–80 ppm) yield greater reductions and consistently achieve inhibition exceeding 99% across multiple strains [34,37,38]. Consequently, the concentrations selected in the present study (62–248 ppm) were based on the literature and the practical range for “shock” treatment recommended to eliminate biofilms and pathogens (40–80 ppm).

In contrast to planktonic cells, biofilm formation exhibited a markedly different behavior when treated with ClO_2_. At 62 ppm ClO_2_, biofilm reduction occurred in serotypes SE and SI, whereas serotype ST exhibited a biphasic response, increasing biofilm formation and showing an adaptive response, in contrast to its sensitivity under similar conditions. This effect suggests that, under these culture conditions, ST is stimulated during biofilm formation, enabling it to adapt to harsh environments and indicating that the ST serotype has a greater ability to adapt and survive under stress [39]. At 124 ppm, biofilm formation decreases across all groups; it decreases most significantly at 248 ppm, with marked reductions observed at 248 ppm within each serotype (Figure 2). However, serotypes SI and SE exhibited more susceptibility at higher concentrations. The differential response among serotypes is particularly relevant, as SI exhibited both the highest baseline biofilm-forming capacity and the greatest susceptibility to ClO_2_ at higher concentrations. This aligns with previous studies [40], indicating that SI is an efficient serotype for biofilm formation, and that pH, nutrients, and temperature influence exopolysaccharide and curli production [41].

For preformed biofilms, treatment with ClO_2_ at 62 and 124 ppm markedly impaired SI biofilm formation, with partial reductions observed for ST and SE. This aligned with reductions of 44.51% and 64.20% in SI at 124 and 248 ppm, respectively, while ST showed smaller reductions (39.72% and 60.70%), and SE displayed variable responses (up to 63.42% at 124 ppm). Corcoran et al. [42] demonstrated the challenge of removing mature biofilms, which are among the least affected by ClO_2_ treatment [43]. Higher concentrations of ClO_2_ (248 and 496 ppm) resulted in marked reductions in biofilm biomass within each serotype, although the magnitude and pattern of response varied among ST, SE, and SI. However, this experiment suggests that serotype SI maintains a more consistent reduction (~64%), while ST and SE exhibit nonlinear responses, indicating possible structural or matrix-dependent protective effects in mature biofilms. The use of ClO_2_ to disinfect eggshells is effective under certain conditions [34], resulting in a significant reduction in bacterial counts for the serotypes evaluated, including SE and SG. However, in the present work, the response to ClO_2_ appeared to be significantly influenced by factors associated with the serotype (ST, SE, and SI), concentration, application method (liquid as opposed to gas), and finally, the presence of organic matter, which is consistent with a previous report [44]. Similarly, previous work has shown that in water-based systems, the primary role of ClO_2_ is often to control microbial load in the surrounding environment rather than to completely eliminate surface-associated bacteria [33]. Finally, studies reinforce the concept that disinfectant performance is highly context-dependent, particularly when comparing aqueous systems with surface or gaseous applications [45].

Differences in gene expression related to oxidative stress, biofilm formation, and energy metabolism among *S. enterica* serovars, along with resistance gene activity and mechanisms such as membrane repair and transport gene overexpression, influence the response to these chemical disinfectants [24,32,46]. Exposure to ClO_2_ affects survival differently across serotypes, with behavioral and adaptive differences among them; this is influenced by several factors observed during the experiment, such as concentration, treatment duration, organic material, metabolites in the TSB medium, and the capacity for adaptation to redox disturbances, efflux pump systems, oxidative stress, and DNA repair among strains, as previously reported with SE [34,47]. While ClO_2_ is considered a promising disinfectant, even in the presence of organic material [17], its effectiveness is affected by higher concentrations of organic material of TSB and higher temperatures (37 °C) during this experiment, as ClO_2_ can be consumed by organic material and degraded by elevated temperatures, reducing its effectiveness under these conditions. These factors are essential for optimizing disinfection strategies across various situations. Factors such as the use of ClO_2_ at high concentrations, prolonged exposure times, moisture, surface type (porosity), and the presence of organic matter are essential considerations [34,48,49].

From another perspective, the efficacy of disinfectants against *Salmonella* varies with serotype, environmental conditions, and the presence of biofilms. Studies indicate that factors such as chemical composition, surface characteristics, and bacterial physiology contribute to this, with cells within biofilms demonstrating greater resistance than planktonic cells [50,51]. Furthermore, susceptibility varies by strain, suggesting that disinfection strategies could benefit from identifying the specific contaminating serovar [52]. From a practical perspective, it is not necessary to identify *Salmonella* serovars before applying these findings, as the results provide a framework for selecting disinfectant concentrations effective against tolerant responses under experimental conditions. This method aligns with actual biosecurity practices—where disinfectants are used without identifying the specific strain—thereby ensuring their efficacy across various contamination scenarios.

ClO_2_ is a promising disinfectant, but environmental conditions affect its performance, underscoring the need for a comprehensive approach to *Salmonella* control in diverse ecological settings [53]. We demonstrated a serovar-dependent effect of ClO_2_ on growth and biofilm formation, with SI showing the highest susceptibility, SE showing an intermediate response, and ST showing comparatively greater tolerance depending on the condition evaluated. Although general patterns were observed, responses varied depending on serotype and experimental condition, particularly in biofilm-related assays. Furthermore, biofilm formation was more pronounced in SI without chemical treatment. These findings highlight the importance of optimizing disinfection strategies under realistic environmental conditions, where variability in serotype behavior and biofilm formation may significantly impact control efficacy.

## Figures and Tables

**Table 1:** Effect of chlorine dioxide (ClO_2_) at concentrations of 62, 124, and 248 ppm on the in vitro proliferation of *S. enterica* serovars Typhimurium (ST), Enteritidis (SE), and Infantis (SI). Results are presented as CFU/mL values, percentage change, and fold change relative to the non-treated control.

Serotype	ClO_2_ (ppm)	CFU/mL	% Change vs. Control *	Fold Change *
ST	Non-treated control	1.67 × 10^9^	-	1.00
ST	62	1.43 × 10^9^	−14.4%	0.86
ST	124	6.92 × 10^8^	−58.6%	0.41
ST	248	3.30 × 10^7^	−98.0%	0.02
SE	Non-treated control	1.87 × 10^9^	-	1.00
SE	62	2.06 × 10^9^	+10.2%	1.10
SE	124	1.59 × 10^7^	−99.1%	0.01
SE	248	3.04 × 10^7^	−98.4%	0.02
SI	Non-treated control	1.42 × 10^9^	-	1.00
SI	62	1.34 × 10^9^	−5.6%	0.94
SI	124	2.02 × 10^2 †^	−99.9%	0.01
SI	248	0	−100%	0.00

* Percentage change and fold change were calculated relative to the non-treated control for each serovar (ST, SE, and SI) using back-transformed CFU/mL values derived from Log_10_ CFU/mL measurements. ^†^ At 124 ppm, serotype SI exhibited minimal detectable bacterial growth (~10^2^ CFU/mL) in 2 out of 7 replicates, while recovery from the remaining replicates was below detectable limits. Under these conditions, percent reduction and fold change are presented as approximate values to reflect complete or near-complete inhibition of bacterial growth.

**Table 2:** Effect of chlorine dioxide (ClO_2_) at concentrations of 62, 124, and 248 ppm on biofilm formation by *Salmonella enterica* serovars Typhimurium (ST), Enteritidis (SE), and Infantis (SI) under in vitro conditions. Biofilm biomass was quantified by crystal violet staining and measured as optical density at 600 nm (OD600). Results are expressed as percentages and fold changes relative to the non-treated control.

Serotype	ClO_2_ (ppm)	OD600	% Change in OD600 for Treatment Compared to Non-Treated Control *	Fold Change *
ST	Non-treated control	0.2186	-	1.00
ST	62	0.3308	+51.32%	1.51
ST	124	0.1136	−48.03%	0.52
ST	248	0.0388	−82.24%	0.18
SE	Non-treated control	0.2372	-	1.00
SE	62	0.1410	−40.55%	0.59
SE	124	0.1001	−57.80%	0.42
SE	248	0.0137	−94.23%	0.06
SI	Non-treated control	0.3721	-	1.00
SI	62	0.1695	−54.44%	0.46
SI	124	0.0409	−89.00%	0.11
SI	248	0.0635	−82.93%	0.17

Biofilm formation was quantified using crystal violet staining (1%) and measured as OD600. * Percentage change and fold change were calculated relative to the non-treated control for each serovar (ST, SE, and SI). Fold change was calculated as the ratio of treated to control OD600 values, and percentage change was derived accordingly.

**Table 3:** Effect of chlorine dioxide (ClO_2_) at concentrations of 62, 124, 248, and 496 ppm on the elimination of preformed biofilm of *Salmonella enterica* serotypes Typhimurium (ST), Enteritidis (SE), and Infantis (SI), expressed as percentage and fold change.

Serotype	ClO_2_ (ppm)	OD600	% Change in OD600 for Treatment Compared to Non-Treated Control *	Fold Change *
ST	Non-treated control	0.1254	-	1.00
ST	62	0.1253	−0.08%	1.00
ST	124	0.0756	−39.72%	0.60
ST	248	0.0493	−60.70%	0.39
ST	496	0.0649	−48.30%	0.52
SE	Non-treated control	0.1911	-	1.00
SE	62	0.1089	−43.01%	0.57
SE	124	0.0699	−63.42%	0.37
SE	248	0.1072	−43.90%	0.56
SE	496	0.0725	−60.54%	0.38
SI	Non-treated control	0.3051	-	1.00
SI	62	0.2077	−9.21%	0.68
SI	124	0.1693	−44.51%	0.55
SI	248	0.1092	−64.20%	0.36
SI	496	0.1176	−64.45%	0.39

* The percentage change and fold change were calculated with respect to the non-treated control for each serovar (ST, SE, and SI).

## Data Availability

Datasets generated for this study are available from the corresponding author upon request.

## Abbreviations

The following abbreviations are used in this manuscript:

ClO_2_	Chlorine dioxide
ClO_2_^−^	Chlorite ions
EPS	Extracellular polymeric substance
LD50	Lethal Dose 50%
NT	No treatment
OD	Optical density
SE	*Salmonella enterica* Enteritidis
SG	*Salmonella* Gallinarum
XLT-4 agar	Xylose Lysine Tergitol-4 agar
CFU	colony-forming units
SI	*Salmonella enterica* Infantis
ST	*Salmonella enterica* Typhimurium
TSB	Tryptic soy broth
